# Children with severe cerebral palsy during the COVID‐19 pandemic: The vulnerable tell us whether our interventions are working

**DOI:** 10.1111/dmcn.16388

**Published:** 2025-06-14

**Authors:** Nigel Paneth

**Affiliations:** ^1^ Departments of Epidemiology & Biostatistics and Pediatrics & Human Development College of Human Medicine, Michigan State University East Lansing MI USA

## Abstract

**This commentary is on the original article by Reid et al. on pages** 1582–1589 **of this issue.**

The COVID‐19 pandemic required all of us to change our behavior. We were asked to mask, to socially distance, and to wash our hands more scrupulously. And society at large was also charged with behavioral change – restricting border entries and closing down gatherings such as concerts, sports events, and schools. These interventions – often referred to as ‘non‐pharmaceutical interventions’ or ‘physical interventions’ – were variably implemented and often quite unpopular. So it is reasonable to ask, did they do any good?

The venerable leaders of the evidence‐based medicine movement, the Cochrane Collaboration, say no. As is the norm in Cochrane reviews, their review of physical interventions to reduce the spread of respiratory viruses was restricted to randomized controlled trials (RCTs).^1^ These were available for only two aspects of behavioral change: hand hygiene and various forms of masking. The 78 reviewed RCTs did not demonstrate effectiveness, though perhaps hand washing had a modest effect. This pessimistic conclusion, combined with the public disdain for masking and physical distancing, is almost certain to discourage physical interventions when the next pandemic hits, which it inevitably must.

But, if we look at all the evidence about the effects of these interventions on viral transmission and not just RCTs^2^ (most of which, as the Cochrane authors noted, were flawed by lack of compliance and high risk of bias), we might draw different conclusions. For example, reviewing more than 100 observational studies from around the world, Leija‐Martinez et al.^3^ found an 80% reduction in the prevalence of respiratory syncytial virus infections as a cause of childhood hospitalizations after the implementation of non‐pharmaceutical interventions aimed at COVID.

In Porto Alegre, Brazil (a city of more than a million inhabitants), all acute respiratory hospital admissions, including for asthma, pneumonia, and bronchiolitis, were reduced by some 80% with the introduction of COVID lockdowns.^4^ Deriving an index of social distancing from mobile phone signals, they showed a close inverse correspondence between this index and respiratory hospital admissions for children.

Reid et al. show that respiratory hospitalizations in children with severe cerebral palsy (CP) were reduced by 36% during the period of lockdown in Melbourne, Australia.^5^ Their study documents the importance of viral transmission in the hospitalization of children with CP.

What does this tell us?

First, that the preferred method for assessing validity in medicine – the individual patient RCT – provides limited insight into the value of community wide interventions, especially under pandemic conditions. And second, that the effects of these interventions are likely to be most clearly seen in vulnerable populations. Cutting down on the transmission of viral infections will not be noticed by most people. But to children with asthma and to children with severe CP, it clearly makes a world of difference.

## Data Availability

Not required.
